# Severe combined immunodeficiency: improved survival leading to detection of underlying liver disease

**DOI:** 10.1186/s12876-023-02782-8

**Published:** 2023-05-19

**Authors:** Anusha Vittal, Nehna Abdul Majeed, Elizabeth Garabedian, Jamie Marko, David E Kleiner, Rob Sokolic, Fabio Candotti, Harry Malech, Theo Heller, Christopher Koh

**Affiliations:** 1 Clinical Research Section, Liver Diseases Branch, NIDDK, NIH, Bethesda, MD USA; 2 National Human Genome Research Institute, NIH, Bethesda, MD USA; 3 Department of Radiology and Imaging Sciences, NIH, Bethesda, MD USA; 4 Laboratory of Pathology, NCI NIH, Bethesda, MD USA; 5 Translational Hepatology Section, Liver Diseases Branch, NIDDK, NIH, Bethesda, MD USA; 6 IQVIA Biotech, Sharon, MA, MD USA; 7 Division of Immunology and Allergy, University Hospital of Lausanne, Lausanne, Switzerland; 8 National Institute of Allergy and Infectious Diseases, NIH, Bethesda, MD USA

**Keywords:** Adenosine deaminase deficiency, Severe combined immunodeficiency, Liver disease

## Abstract

**Background:**

Adenosine deaminase deficiency (ADA) is an autosomal recessive disorder leading to severe combined immunodeficiency (SCID). It is characterized patho-physiologically by intracellular accumulation of toxic products affecting lymphocytes. Other organ systems are known to be affected causing non-immune abnormalities. We aimed to conduct a cross sectional study to describe liver disease in autosomal recessive ADA-SCID.

**Methods:**

Single center retrospective analysis of genetically confirmed autosomal recessive ADA-SCID was performed. Liver disease was defined as ≥1.5x the gender specific upper limit of normal (ULN; 33 IU/L for males and 25 IU/L for females) alanine aminotransferase (ALT) or moderate and severe increase in liver echogenicity on ultrasound.

**Results:**

The cohort included 18 patients with 11 males. The median age was 11.5 (3.5–30.0 years) and median BMI percentile was 75.5 [36.75, 89.5]. All patients received enzyme replacement therapy at the time of evaluation. Seven (38%) and five (27%) patients had gene therapy (GT) and hematopoietic stem cell transplant (HSCT) in the past. Five patients had 1.5x ALT level more than 1.5x the U. Liver echogenicity was mild in 6 (33%), moderate in 2 (11%) and severe in 2 (11%) patients. All patients had normal Fibrosis-4 Index and Non-alcoholic fatty liver disease fibrosis biomarker scores indicating absence of advanced fibrosis in our cohort. Of 5 patients who had liver biopsies, steatohepatitis was noted in 3 patients (NAS score of 3,3,4).

**Discussion:**

Non-immunologic manifestations of ADA-SCID have become more apparent in recent years as survival improved. We concluded that steatosis is the most common finding noted in our ADA-SCID cohort.

**Supplementary Information:**

The online version contains supplementary material available at 10.1186/s12876-023-02782-8.

## Introduction

Adenosine deaminase deficiency (ADA) is a primary autosomal recessive genetic disorder typically leading to a severe combined immunodeficiency (SCID) [1]. Patients with ADA deficiency have dysfunction of T, B, and natural killer (NK) cells and are identified early in life when they present with recurrent severe infections. It is characterized pathophysiologically by intracellular accumulation of toxic products affecting lymphocytes [2]. There are multiple forms of SCID, namely ADA-SCID, X linked SCID, RAG-1 or RAG-2 SCID and IL7R SCID. The underlying defect of SCID was first identified in ADA-SCID, which accounts for 10–15% of cases of human SCID [3].

The ubiquitous nature of ADA leads to consequences in multiple organ systems including the liver. Improved treatment modalities for SCID patients have revealed non-immunological effects of the disease due to improved survival [1, 4]. Clinical manifestations of ADA-SCID patients include but are not limited to skeletal abnormalities, neurological deficits involving cognitive and motor dysfunction, and hearing loss [5–7].

ADA-SCID is unique compared to other inborn errors of immunity, it affects lymphocyte signaling pathways and is a systemic disorder affecting metabolic pathways. Bollinger et al. reported a case of elevated liver enzymes presenting as neonatal jaundice in a patient with ADA deficiency [8]. To the best of our knowledge, there is no published cohort study of ADA-SCID. The aim of this study was to conduct a cross sectional analysis of liver disease in a cohort of patients with autosomal recessive ADA-SCID.

## Methods

Patients with known or suspected diagnosis of ADA-SCID were referred to the National Human Genome Research Institute at the National Institutes of Health for evaluation for possible hematopoietic stem cell transplant (HSCT) or gene therapy (GT). Patients suspected to have liver disease either due to abnormal liver enzymes or imaging were evaluated and followed prospectively by the Liver Diseases Branch, National Institute of Diabetes and Digestive and Kidney Disease.

We performed a cross sectional retrospective analysis of patients with genetically confirmed ADA-SCID. All subjects provided written informed consent for the study. All patients were part of a natural history clinical research protocol studying the molecular and clinical studies of primary immunodeficiency diseases (NCT00006319) or as part of a clinical research protocol studying the evaluation of liver disease (NCT00001971).

All patients had serum tests including complete blood count (CBC), hepatic panel (including aspartate aminotransferase (AST); alanine aminotransferase (ALT); alkaline phosphatase (ALP); gamma glutamyl transferase (GGT); total bilirubin), creatinine, albumin, international normalized ratio (INR) and lymphocyte proliferation assay (LPA). Ultrasound (US) liver, computed tomography (CT)/magnetic resonance imaging (MRI) and liver biopsy were performed when clinically indicated. Demographic data, clinical events, medications, laboratory results, imaging findings and liver biopsies were recorded. Patient charts were reviewed by two authors (N.A.M, A.V.) to gather data. All laboratory results collected were from samples drawn within 2 weeks of the imaging studies. We defined liver disease as 1.5 times the gender specific upper limit of normal (ULN) of (ALT) and/or moderate or severe increase in liver echogenicity on US.

### Liver enzymes

Liver enzyme elevations, particularly alanine aminotransferase (ALT), was recorded as a fraction of the gender related upper limit of normal. 25 U/L and 33 U/L were used as the upper limit of normal (ULN) for ALT for women and men, respectively [9]. Fraction of age related ULN for ALP, GGT, liver size and spleen size were used for analysis [9], [10].

#### Immunology

Lymphocyte proliferation assay (LPA) was utilized to measure the ability of lymphocytes to undergo clonal proliferation after stimulation. Phytohemagglutinin (PHA) 2.5 µg/ml or 5 µg/ml were utilized to analyze response at 3 days and tetanus toxoid 1 µg/ml at 6 days. Stimulation indices were analyzed Adenosine deaminase (ADA) levels were not analyzed as only one patient in the cohort had the level measured within two weeks of the imaging studies.

### Imaging

Imaging of the liver included in this analysis are ultrasound (US), computed tomography (CT) or magnetic resonance imaging (MRI). CT and MRI were performed for further evaluation of abnormal findings such as a lesion noted on the US liver. All the US, MRI and CT images were reviewed by a single radiologist (J.M). Liver size, contour, echogenicity, portal vein flow and velocity, and spleen size were recorded for US images. CT and MRI were used to look for collateral formation, varices, splenorenal shunt formation, recanalization of paraumbilical vein and to characterize any hepatic lesions seen on US. Fraction of age-related ULN of liver and spleen size were recorded to avoid age being a confounder.

### Liver biopsy

Liver biopsy was performed when clinically indicated, i.e., unclear etiology of liver disease or persistent elevation of liver enzymes. Liver biopsies were performed percutaneously. Eight patients had liver biopsy and all of them were reviewed by the same hepatopathologist (D.E.K.). All the biopsies were scored for steatosis, lobular inflammation, hepatocellular ballooning and fibrosis [11].

### Statistical analysis

Statistical analysis was performed using GraphPad Prism Version 8.1.1. Descriptive statistics of baseline demographic data were presented as frequencies for categorical variables and mean with standard deviation for continuous variables. Comparison of clinical and biochemical characteristics of patients with and without liver disease was performed using Mann-Whitney test for non-parametric continuous variables. A p value < 0.05 was considered as statistically significant.

Patients were categorized into four groups based on the severity of echogenicity, namely, normal, mild, moderate, and severe increase in echogenicity [12]. All the values were arranged in ascending order and plotted on a graph with X-axis denoting the patient code and Y axis-denoting the variable using Microsoft Excel for Mac, Version 16.52.

## Results

Between January 2006 to June 2019, 18 patients out of the 23 patients with ADA-SCID, seen at NIH, were included in the analysis. Five patients were excluded due to incomplete records, lab data or imaging studies.

### Patient characteristics

Patient characteristics are described in Table 1. The median age when seen was 11.5 years, (range 3.5 to 30.0 years). There were 11 males (61% of the cohort), and the median BMI of the cohort was 18.4. The cohort included 10 White, 5 African American, 1 Native American, 1 Multiracial and 1 patient with unknown race. Seven (38%) and five (27%) patients had unsuccessful gene therapy (GT) and hematopoietic stem cell transplant (HSCT) in the past, respectively. All the patients were receiving enzyme replacement therapy at the time of evaluation. All patients had US of liver and five (27%) had liver biopsies.

**Table 1:** Patient characteristics

Number of patients in the cohort (n)	18
Median age (years)	11.5(3.5–30.0)
Males [n (%)]	11 (61%)
BMI percentile (median)	75.5
Race [n(%)]	
White	10 (55%)
African-American	5 (27%)
Native American	1 (5%)
Multiracial	1 (5%)
Unknown	1 (5%)
Gene Therapy [n(%)]	7 (38%)
HSCT [n(%)]	5 (27%)
ERT [n(%)]	18 (100%)

HSCT: hematopoietic stem cell transplantation; ERT: Enzyme replacement therapy; BMI: Body mass index

### Biochemical pattern of liver disease

**Fig. 1:**
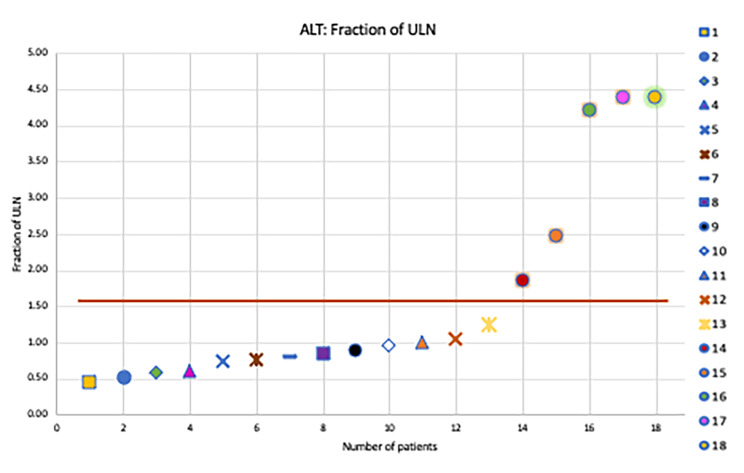
ALT levels charted as fraction of the gender related ULN. X-axis: Serial number. Y-axis: Fraction of gender related ULN for ALT. Each patient is coded with a specific shape, shown on the right of the graph. Patients 14, 15, 16, 17 and 18 have elevated liver enzymes (> 1.5x ULN)

**Fig. 2:**
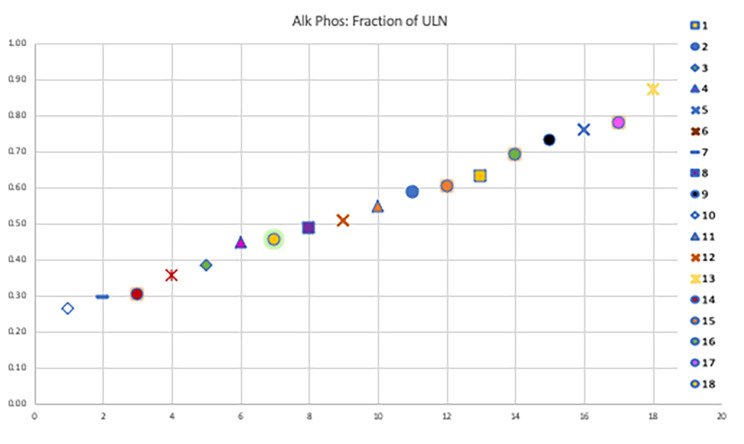
ALP levels charted as fraction of the age-related ULN. X-axis: Serial Number. Y-axis: Fraction of the age related ULN. Each patient is coded with a specific shape, shown on the right of the graph

Liver enzymes were analyzed by charting fraction of gender related ULN. On analysis of ALT, it was noted that five patients (Patients 14, 15, 16, 17 and 18) had ALT levels more than 1.5x the gender related ULN. Patient 14 had 1.7x, Patient 15 had 2.5x, Patient 16 had 4x, Patient 17 had 4.1x and patient 18 had 4.4x the gender related ULN for ALT(Fig. 1). Patient 18 who had 4.4x elevation in ALT was thought to be due to drug induced liver injury from chart review. Patient 18 was found to have hepatocellular pattern injury a week after initiating amoxicillin-clavulanate for sinus infection. All patients had normal levels of alkaline phosphatase as shown in Fig. 2.

### Comparison of characteristics of patients with liver disease and no liver disease

Characteristics of patients with and without liver disease based on ALT elevation were compared and significant differences were found in BMI, ALT, GGT and liver size (Table 2). No additional differences between laboratory or demographic characteristics were identified between the groups. Given the small cohort, we could not identify potential predictors for development of liver disease.

**Table 2:** Comparison of characteristics between patients with liver disease and no liver disease

Parameter	Liver disease (n = 5)	No liver disease (n = 13)	P-value
Age, years	13.6±6.7	11.8±7.4	0.38
Males [n]	3	8	
BMI, kg/m^2^	26.6±7.9	18.9±4.4	0.046*
ALT	3.5±1.2	0.8±0.2	0.0002*
ALP	0.6±0.2	0.5±0.2	0.68
T.Bil	0.2±0.1	0.2±0.03	0.5
GGT	3.3±2.6	1.0±0.4	0.0003*
INR	1.0±0.1	1.0±0.05	0.12
Albumin	3.9±0.6	4.2±0.4	0.48
Liver size	1.4±0.2	1.1±0.1	0.02*
Spleen size	0.8±0.2	0.8±0.1	0.79
Creatinine, mg/dL	0.6±0.1	0.4±0.1	0.16
Platelets, k/µL	300±101	245±80	0.40

*p-value less than 0.05 is significant

Liver enzymes are plotted as fraction of age related ULN.

Variables are denoted as mean with standard deviation except the variable male

**Fig. 3:**
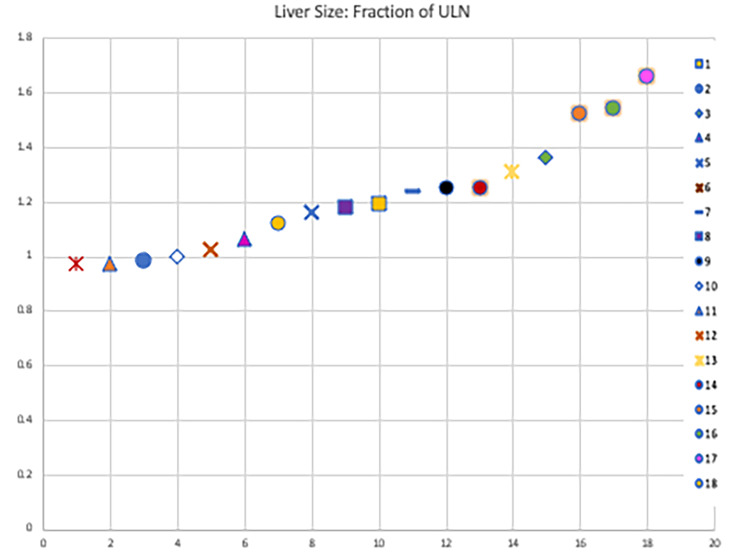
Graph plot of liver size. X-axis: Serial numbers. Y-axis: Fraction of age related ULN of liver size. Each patient is coded with a specific shape, shown on the right of the graph

### Immunology

The 3-day proliferation of lymphocytes after stimulation with PHA 2.5 µg/ml or 5 µg/ml did not show a statistically significant difference between the groups. Similarly, the 6-day proliferation after tetanus toxoid 1 µg/ml was not statistically significant between the groups.

### Imaging

**Fig. 4:**
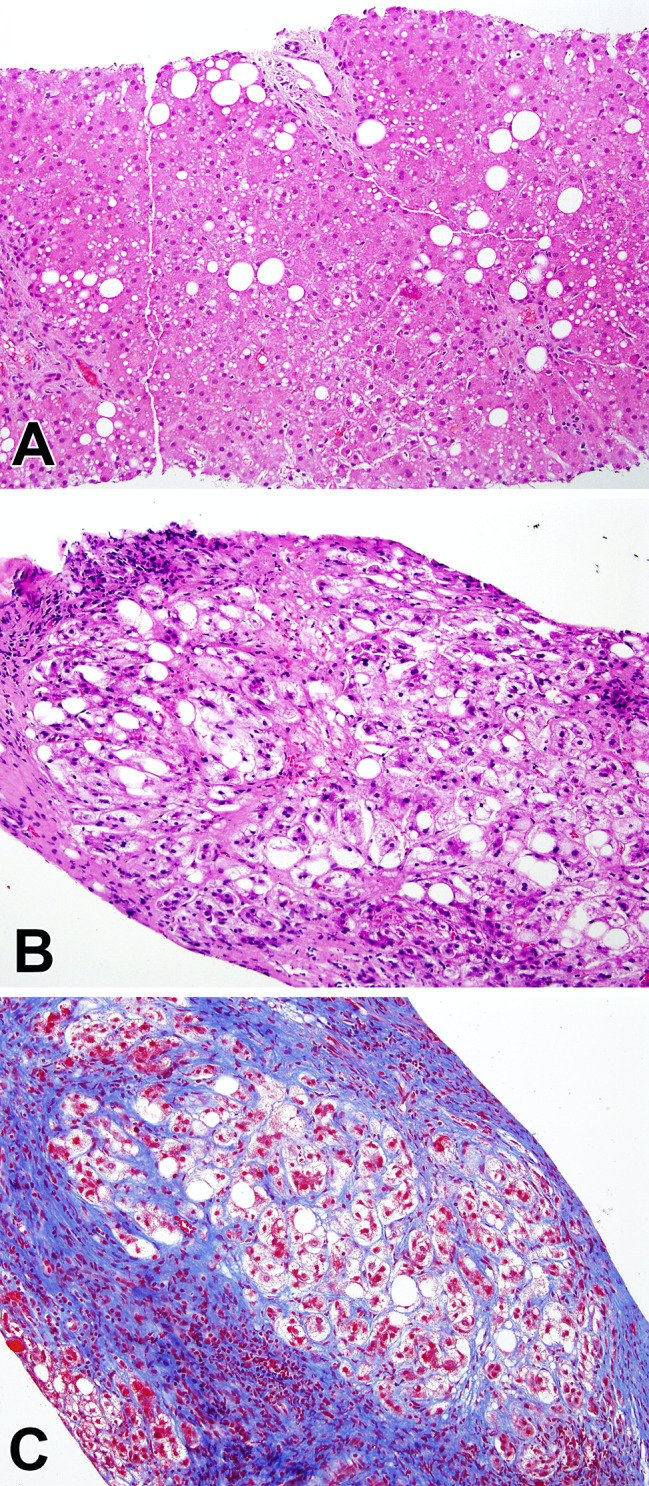
Fatty liver disease in SCID. A. Mild steatosis near the portal area in a zone 1 distribution (H&E, 200x). B, C. Steatohepatitis with focally advanced fibrosis. (B: H&E, 200x; C: Masson Trichrome, 200x)

Of the 18 patients followed, all patients had ultrasound of liver and spleen. Seven patients had CT scans and six patients had MRI of abdomen. Patients were categorized into four groups based on the severity of echogenicity, namely, normal, mild, moderate, and severe increase in echogenicity. Ten of the eighteen patients (55%) had steatosis on liver ultrasound, of these 6 (33%) patients had mild increase in echogenicity, 2 (11%) patients had moderate and 2 (11%) had severe increase in echogenicity (Fig. 4). One patient (5%) was also noted to have coarsened echotexture of the liver. Five of the 6 patients (83%) with mild increase in echogenicity on ultrasound, had normal liver enzymes and isolated abnormalities on liver US. All patients with moderate and severe increase in echogenicity had elevation of ALT and GGT. Two patients were observed to have an increase in spleen size for age, patients 13 and 15. All patients had normal portal flow, portal flow velocity and surface contour of liver. All patients except patient 6 were noted to have normal liver echotexture. Patient 6 had mildly coarsened liver echotexture. Ultrasound findings were corroborated by CT and MRI.

### Categorization of patients based on liver echogenicity on US liver

Patients 16 and 18 had moderate increase in echogenicity and Patients 14 and 17 had severe increase in echogenicity. Patients with increase in echogenicity were also noted to have increased liver size, recorded as fraction of the age related ULN. Patients 13 and 17 with severe increase in echogenicity had 1.25x and 1.54x the ULN of liver size for age, respectively. Patients 16 and 18 with moderate increase in echogenicity had 1.52x and 1.66x the ULN of liver size for age, respectively (Table 2; Figs. 2 and 3).

### Role of non-invasive markers in diagnosis of ADA-SCID liver disease

We calculated noninvasive fibrosis markers such as AST-to-platelet-ratio-index (APRI), Fibrosis-4 index (FIB-4), and NAFLD fibrosis score. All 18 patients had normal FIB-4 and NAFLD fibrosis score indicating absence of advanced fibrosis in our cohort (Supplementary Table 1). Of four patients who had abnormal APRI score, 2 of them (Patients 18 and 13 had histological evidence of peri-portal and bridging fibrosis respectively). Patients 8 and 16 with abnormal APRI score had no and severe steatosis respectively.

### Histological characteristics

Seven patients underwent liver biopsy and one (patient 6) underwent a resection for hepatoblastoma (Table 3). The two youngest patients (5 and 6) in this subgroup had no pathological changes (aside from the hepatoblastoma in patient 6). The remaining biopsies all showed steatotic liver disease. Of this group of six, 4 had generally mild macrovesicular steatosis, distributed predominantly in zone 1, associated with mild portal and lobular inflammation (Fig. 4A). Fibrosis, present in three, was periportal, with one case also showing perisinusoidal fibrosis around the vein. The other two patients had steatohepatitis, with mild ballooning injury in addition to mild to moderate steatosis and mild inflammation (Fig. 4B). Advanced bridging fibrosis was seen in one (Fig. 4C), while the other only showed periportal and perisinusoidal fibrosis. There was no evidence of nodular regenerative hyperplasia in any of the cases.

**Table 3:** Histological characteristics

Patient	Characteristic	NAS		Fibrosis Stage	Steatosis Grade	Ballooning Grade
3	Steatohepatitis		3	2-PPF + PSF	1	1
5	Normal liver parenchyma					
6	Hepatoblastoma, normal uninvolved liver parenchyma					
7	Steatosis		1	0-NF	1	0
11	Steatosis		2	1-PPF	1	0
15	Steatohepatitis		4	3-BF	2	1
17	Zone 1 borderline		3	3-BF	2	0
18	Zone 1 borderline		3	2-PPF + PSF	2	0

NAS: NAFLD activity score

NF: No fibrosis

PPF: Periportal fibrosis

PSF: Perisinusoidal fibrosis

BF: bridging fibrosis

1: PPF

2: PPF + PSF

## Discussion

In this single-center retrospective cross sectional study, we report for the first time the liver disease in the largest cohort of patients with ADA-SCID. 18 patients with ADA SCID with median age of 11.5 (3.5–30.0 years) were included. With recent advances in treatment options and improvement in patient care, the overall survival of patients with SCID has improved and non-immunologic manifestations of ADA-SCID have become more apparent. With this success, identifying complications affecting the liver, was possible as the likelihood of organ involvement increases with longer lifespans.

All patients received enzyme replacement therapy, 7 of them had gene therapy and five of them received stem cell transplant. Five patients were noted to have liver disease based on elevated ALT, more than 1.5x the gender specific ULN. Of which one patient was thought to have drug induced liver injury (DILI) based on chart review. All the patients with elevated liver enzymes had moderate or severe increase in liver echogenicity on US except the patient with DILI. One patient who had evidence of moderate steatosis on US was noted to have steatohepatitis measured by histopathology NASH-CRN score. Characteristics found to be statistically significant and different between the group with liver disease and no liver disease were BMI, ALT, GGT and liver size. Patients with liver disease were noted to be overweight (BMI 26.6), have higher ALT (4.2x gender specific ULN), GGT (2.7x the age related ULN) and liver size (1.5x the age related ULN).

There have been case reports of infants with ADA-SCID presenting with acute hepatitis within weeks of birth [8]. In adenosine deaminase deficiency, intra-cellular accumulation of deoxyadenosine triphosphate was the cause of lymphopenia [13, 14]. The level of deoxyadenosine triphosphate in hepatocytes was minimal. Increased level of inactivated S-adenosylhomocysteine hydrolase (SAH) enzyme causing causing hepatic accumulation of the toxic metabolite, adenosylhomocysteine [15, 16]. This was postulated to be the cause of hepatitis in these patients. The case report by Bollinger et al. in 1996, presented with hepatitis and was treated with enzyme replacement. Patient had normalization of liver enzymes likely due to resolution of toxic metabolite accumulation as mentioned above. A case series was published by Ratech and et al. in 1985 about the autopsy findings of 8 patients who had ADA-SCID. Two out of the 8 had elevated liver enzymes, no infectious causes identified and autopsy showed bridging fibrosis [17]. We think the liver disease observed in our cohort of ADA-SCID patients is different from that described in the past. Patients in our cohort were diagnosed to have ADA-SCID early and all of them were started on enzyme replacement early in life. Therefore, it is unlikely that our ADA-SCID cohort patients had toxic accumulation of metabolites as there are no reports of hepatitis in the first few months of life. The most common liver disease noted in our cohort was steatosis and these patients had multiple risk factors including multiple infections, exposure to medications and elevated BMI. Another possible mechanism of steatosis in this population includes dysregulation of metabolic interconnectivity observed in immune cell biology. T cell mediated immunity requires stimulation of insulin mediated receptors [18]. SCID patients lack T cells, causing reduction in insulin uptake. This leads to elevated levels of insulin in circulation thereby causing activation of glycogen synthesis [19].

In terms of limitations, this is a retrospective cross-sectional study with a small cohort of patients. However, our cohort is the largest ADA-SCID cohort reporting liver disease for the first time. Another limitation of our study is lack of longitudinal data as most of our patients were lost to follow up. Also, very few patients who had suspicion of liver involvement underwent liver biopsies for diagnostic purposes. Another limitation of our study is inability to explore the correlation of liver injury with ERT and gene therapy due to its cross sectional data set and missing some data points.

ADA-SCID patients are now surviving into adulthood with major advances in medical treatment thus uncovering long term effects of ADA deficiency in non-immunological organs including liver. Early diagnosis and initiation of treatment with enzyme replacement is key in preventing acute hepatitis from toxic accumulation of products from purine metabolism. However, the risk of developing other forms of liver disease in this population still exists. One of the reasons is increased risk of infections and exposure to multiple medications in their lifetime as most of the patients receive either HSCT or GT. There is also a possibility of partial immune reconstitution due to no engraftment of B cells which is clinically similar to common variable immunodeficiency. This increases their risk of developing liver disease such as nodular regenerative hyperplasia [20].

In conclusion, we propose that steatosis is the most common finding noted in our ADA-SCID cohort. ADA-SCID patients are at increased risk for developing chronic liver disease and warrant close monitoring to help prevent onset or progression of disease as most of the patients in our cohort are young.

## Electronic supplementary material

Below is the link to the electronic supplementary material.

Supplementary Table 1: Non-invasive markers of all patients

## Data Availability

All datasets used and/or analysed during the current study available from the corresponding author on reasonable request.

## Abbreviations

ADA- SCID: Adenosine deaminase deficiency - Severe combined immunodeficiency
ERT: Enzyme replacement therapy
GT: Gene therapy
HSCT: Hematopoietic stem cell transplant
