# Supplementary material for: Severe combined immunodeficiency: improved survival leading to detection of underlying liver disease
Source: BMC Gastroenterol. 2023 May 19;23:166. doi: 10.1186/s12876-023-02782-8 (PMC10199556; doi:10.1186/s12876-023-02782-8)
Supplement: Supplementary file 1 — Supplementary Table 1: Non-invasive markers of all patients [file 12876_2023_2782_MOESM1_ESM.docx]

**Supplementary Table 1: Non-invasive markers of all patients**

| Patient Number | APRI | NAFLD Fibrosis Score | FIB-4 |
| --- | --- | --- | --- |
| 1 | 0.3 | -4.0 | 0.16 |
| 2 | 0.3 | -4.9 | 0.19 |
| 3 | 0.3 | -2. | 0.36 |
| 4 | 0.2 | -4.3 | 0.15 |
| 5 | 0.5 | -4.3 | 0.16 |
| 6 | 0.2 | -6.3 | 0.05 |
| 7 | 0.2 | -2.9 | 0.52 |
| 8 | 0.7 | -2.6 | 0.88 |
| 9 | 0.1 | -6.4 | 0.22 |
| 10 | 0.5 | -2.5 | 0.59 |
| 11 | 0.6 | -4.0 | 0.47 |
| 12 | 0.3 | -5.2 | 0.08 |
| 13 | 0.3 | -5.0 | 0.19 |
| 14 | 0.4 | -3.9 | 0.39 |
| 15 | 0.8 | -1.6 | 0.82 |
| 16 | 1.2 | -4.3 | 0.67 |
| 17 | 0.4 | -7.9 | 0.05 |
| 18 | 0.8 | -4.4 | 0.23 |
